# *GridSample*: an R package to generate household survey primary sampling units (PSUs) from gridded population data

**DOI:** 10.1186/s12942-017-0098-4

**Published:** 2017-07-19

**Authors:** Dana R. Thomson, Forrest R. Stevens, Nick W. Ruktanonchai, Andrew J. Tatem, Marcia C. Castro

**Affiliations:** 1 Department of Social Statistics and Demography, University of Southampton, Building 58, Southampton, SO17 1BJ UK; 2 WorldPop, Department of Geography and Environment, University of Southampton, Building 44, Southampton, SO17 1BJ UK; 3 Flowminder Foundation, Roslagsgatan 17, 11355 Stockholm, Sweden; 4 Department of Geography and Geosciences, University of Louisville, 200 E Shipp Ave, Louisville, KY 40208 USA; 5 Department of Global Health and Population, Harvard T.H. Chan School of Public Health, 665 Huntington Ave, Boston, MA 02115 USA

**Keywords:** Cluster survey, Multi-stage, Cluster sample

## Abstract

**Background:**

Household survey data are collected by governments, international organizations, and companies to prioritize policies and allocate billions of dollars. Surveys are typically selected from recent census data; however, census data are often outdated or inaccurate. This paper describes how gridded population data might instead be used as a sample frame, and introduces the R *GridSample* algorithm for selecting primary sampling units (PSU) for complex household surveys with gridded population data. With a gridded population dataset and geographic boundary of the study area, *GridSample* allows a two-step process to sample “seed” cells with probability proportionate to estimated population size, then “grows” PSUs until a minimum population is achieved in each PSU. The algorithm permits stratification and oversampling of urban or rural areas. The approximately uniform size and shape of grid cells allows for spatial oversampling, not possible in typical surveys, possibly improving small area estimates with survey results.

**Results:**

We replicated the 2010 Rwanda Demographic and Health Survey (DHS) in *GridSample* by sampling the WorldPop 2010 UN-adjusted 100 m × 100 m gridded population dataset, stratifying by Rwanda’s 30 districts, and oversampling in urban areas. The 2010 Rwanda DHS had 79 urban PSUs, 413 rural PSUs, with an average PSU population of 610 people. An equivalent sample in *GridSample* had 75 urban PSUs, 405 rural PSUs, and a median PSU population of 612 people. The number of PSUs differed because DHS added urban PSUs from specific districts while *GridSample* reallocated rural-to-urban PSUs across all districts.

**Conclusions:**

Gridded population sampling is a promising alternative to typical census-based sampling when census data are moderately outdated or inaccurate. Four approaches to implementation have been tried: (1) using gridded PSU boundaries produced by *GridSample*, (2) manually segmenting gridded PSU using satellite imagery, (3) non-probability sampling (e.g. random-walk, “spin-the-pen”), and random sampling of households. Gridded population sampling is in its infancy, and further research is needed to assess the accuracy and feasibility of gridded population sampling. The *GridSample* R algorithm can be used to forward this research agenda.

**Electronic supplementary material:**

The online version of this article (doi:10.1186/s12942-017-0098-4) contains supplementary material, which is available to authorized users.

## Background

Household survey data are collected to support prioritization of national and international issues, allocate billions of donor and government dollars, track progress toward major policy and program goals including the sustainable development goals (SDGs) [1, 2], quantify needs during disaster responses [3, 4], and follow consumer trends [5]. Household surveys are particularly important in countries where census data, or other forms of official data such as birth and death registries, are outdated, incomplete or inaccurate. Selection of representative household survey samples requires definition of areal units with up-to-date and accurate population counts—typically enumeration areas from a recent census—creating a circular dilemma. Where census data are not available, outdated, or known to be unreliable, individual survey teams have begun to experiment with gridded population sampling as an alternative [6–11], and organizations that fund routine surveys are beginning to recommend gridded population datasets as alternative sample frames [12]. To date, however, no tools exist to support complex survey selection from gridded population datasets, and there is scant guidance to use these emerging methods. This paper (1) describes how gridded population datasets have been used as alternative sample frames to outdated or inaccurate census data, (2) introduces *GridSample* [13], an R package, for the first-stage selection of complex household surveys using gridded population data, and (3) summarizes options to implement gridded population samples in the field. R is an open-source free software environment created and maintained by hundreds of developers from many disciplines worldwide. R contains well-established, user-created packages to perform statistical analysis and data visualization.

### Typical household surveys

Since the 1980s, hundreds of nationally-representative household surveys have been conducted by governments in low- and middle-income countries roughly every five years with support from the United Nations (UN) [14, 15], the US Government [16], and the World Bank [17] to monitor social, demographic, economic, and health indicators. The UN’s Multiple Indicator Cluster Surveys (MICS), the US Government’s Demographic and Health Surveys (DHS), and the World Bank’s Living Standard Measurement Surveys (LSMS) stratify samples by sub-national region, and sample roughly 10,000 households in a two-stage design that is widely used by survey implementers to maximize statistical power and feasibility while minimizing costs and potential biases [14–16]. Each of these surveys cost several hundred thousand US dollars and approximately two years to implement and publish [18].

**Fig. 1:**
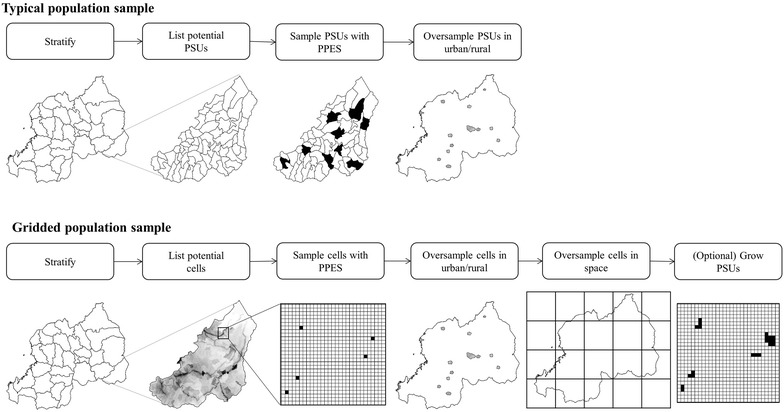
Comparison of first stage in typical population sampling and gridded population sampling

In standard large-scale household surveys, implementers sample communities first (called clusters, or primary sampling units—PSUs) from recent census enumeration areas. Then second, list all households in the sampled communities during a field mapping exercise before systematically sampling households [13, 15, 16] (Fig. 1). In the poorest settings, household enumeration is still routinely performed by hand with a pencil and paper [16], and satellite-enhanced enumeration has been piloted with printed maps of satellite imagery and with mobile devices [19, 20]. While these methods are widely adopted and considered the gold-standard, they are limited in their ability to generate accurate samples when census data frames are outdated or inaccurate [21]. At the time of this writing in 2017, 37 of 157 countries in Africa, Asia, and Central and South America has a census that is 10 years old or more [22]. Many of these countries have experienced population displacement by environmental disasters, conflict, rapid economic change [23], official changes to subnational administrative area boundaries [24] and normal demographic shifts due to changing birth and death rates.

### Gridded population data

Gridded population data may prove to be a viable alternative sample frame where census data are outdated or inaccurate. Three types of gridded population datasets are available. First, standard “top-down” gridded population datasets are generated by models that either directly disaggregate administrative population counts to grid cells using satellite imagery (e.g. land cover and nighttime lights) and other spatial data (e.g. road and building locations), or non-uniformly disaggregate population counts using complex modeling approaches. Direct disaggregation approaches vary from simple areal weighting (e.g. GPWv4 [25], UNEP [26]) to use of ancillary data, such as urban settlement areas, to inform the location and density of disaggregated population (e.g. GRUMP [27], GHS-Pop [28], Facebook [29]). Complex modelling techniques (e.g. WorldPop [30], Landscan [31], Demobase [32]) include such methods as aggregating input and covariate data at two scales to test and tailor the model to local areas.

Multiple top-down global gridded population datasets are available to freely download including WorldPop [33], GPWv4 [34], GHS-POP [35], GRUMP [36], and UNEP [26]. Landscan [37] datasets are free to US Federal Government agencies and some humanitarian, education and commercial organizations, upon request. Gridded population datasets are published as population estimates per pixel, where pixels are measured in decimal degrees and are thus slightly smaller and less square-shaped toward the earth’s poles compared to the equator. Within countries, differences in cell size are generally negligible; exceptions include Brazil and Russia with large north–south coverage. WorldPop [33] additionally provides population per hectare estimates measured in meters, where each pixel is 100 m × 100 m anywhere on earth. Gridded population datasets have known inaccuracies, particularly at the sub-national and metropolitan scales [38, 39]. Although top-down gridded population datasets may be based on outdated or incorrect population totals from 2nd-, 3rd-, and 4th-level administrative areas, the distribution of population estimates within administrative areas might be more representative of the population than enumeration area counts in the last census.

Gridded population data need not be based entirely on census data. Where census data are grossly outdated and populations are reasonably stationary, researchers are experimenting with a second type of gridded population dataset using “bottom-up” methods that integrate population counts from small area surveys with dozens of spatial covariates [40]. In areas where large-scale population movement has resulted from a major event, such as an earthquake or violent conflict, researchers have begun to work with mobile phone companies to gain anonymized, aggregated call detail records (CDR) and generate a third type of CDR-enhanced gridded population dataset [41–43].

### Gridded population sampling for household surveys

The *GridSample* package was recently released in R CRAN to generate PSUs for household surveys using gridded population data rather than census data [13]. *GridSample* supports typical complex sample designs including stratification, oversampling in urban or rural areas, and sampling of different numbers of households within urban and rural areas (Fig. 1). Because grid cells are approximately uniform in size and shape within a country, *GridSample* also allows for a population sample to be supplemented with a spatial oversample in remote areas which is attractive if survey results will be used to generate small area estimates or make interpolated surfaces [44] (Fig. 1).

The user needs either two or three datasets to use *GridSample*. First, a gridded population dataset that covers the study area. Gridded population data are produced in raster file format. A common example of a raster dataset is a photograph which is comprised of pixels, each with a single color value. Similar to a photograph, gridded population cells each have one estimated population value. Second, the user provides the boundary of the study area if the sample is not stratified, or boundaries of geographic strata if the sample is stratified. Third, the user optionally inputs urban/rural area boundaries if urban and rural domains will be represented in the survey. Boundaries are commonly formatted as a shapefile, a type of file used to store points, lines, or polygons (areas) and their attributes. *GridSample* requires that all input datasets are converted to raster format using the same grid cell dimensions as the population dataset. Below, we provide a code example to convert shapefiles to rasters.

**Fig. 2:**
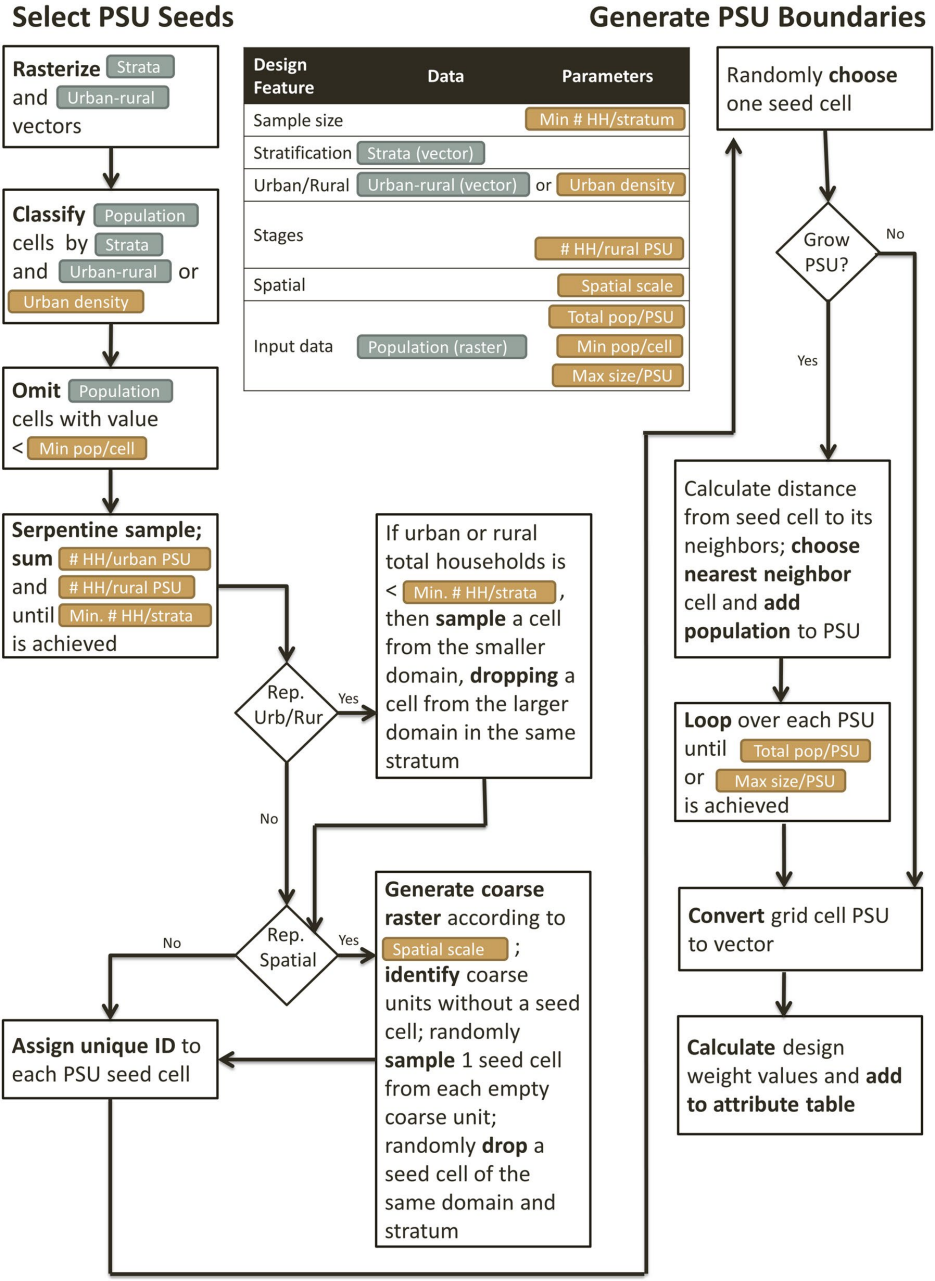
*GridSample* workflow

The input raster datasets, plus a number of survey parameters, are used to randomly sample grid cells with probability proportionate to estimated population (PPES) size in a first step, and then optionally grow PSUs around selected seed cells until a minimum population threshold is achieved in a second step (Fig. 2). This two-step process ensures both that the desired number of PSUs per strata and domain are achieved, as well as desired population per PSU. *GridSample* outputs a shapefile of PSU boundaries which can be visualized in a geographic information system like QGIS™ or ArcGIS™, or overlaid on satellite imagery, for example in Google Earth™. The shapefile includes a record for each PSU containing the latitude-longitude coordinate of the PSU centroid (geographic center), and the PSU and strata population counts needed in sample weight calculations.

In the following sections, we provide a technical overview of the *GridSample* algorithm workflow; describe how to replicate typical complex survey designs in *GridSample*; describe the use of population sampling with a spatial oversample; and reproduce an existing DHS sample in *GridSample*. To support use of *GridSample*, we provide sample weight calculation instructions, discuss practical limitations, outline areas for future gridded population survey research, and offer suggestions to improve the feasibility of fieldwork.

### *GridSample*: technical workflow

*GridSample* is an R CRAN package with four functions—gs_mode, gs_rasterize, gs_zonal_raster, and gs_sample—though the user only interacts with the main function, gs_sample. *GridSample* is written for R version 3.2.3 or newer, and requires the following libraries: *rgdal* (≥1.2–5), *raster* (≥2.5–8), *data.table* (≥1.10.4), *rgeos* (≥0.3–22), *geosphere* (≥1.5–5), *sp* (≥1.2–4), *deldir* (≥0.1–12), *spatstat* (≥1.49–0), and *maptools* (≥0.8-41). Figure 2 visualizes how the input datasets and parameters are processed in gs_sample. At a minimum, the user must specify the input gridded population dataset (population_raster), household sample size (cfg_hh_per_stratum), study area boundary (which is strata_raster, the boundary of a single stratum sample), population size per PSU (cfg_pop_per_psu), and number of households to be sampled per PSU (the urban value cfg_hh_per_urban is used for all PSUs if a rural value cfg_hh_per_rural is not specified). Further complexities can be added to the survey design including stratification, oversampling of urban/rural populations, and spatial sampling. *GridSample* first selects PSU seed cells from the dataset, and then optionally grows each PSU by adding neighboring cells until a minimum geographic size (cfg_max_psu_size) or population size (cfg_pop_per_psu) is achieved.

Before using gs_sample, the user must rasterize all vector data to match the grid dimensions of the gridded population dataset (population_raster). Specifically, the user must rasterize urban/rural boundaries and strata boundaries. Urban/rural boundaries (urban_raster) may be defined from existing data sources such as Global Urban Footprint (GUF) [45], Global Rural Urban Mapping Project (GRUMP) [36], Global Human Settlement City Model (GHS-SMOD) [46], Modis 500 m urban extents [47], and European Space Agency Land Cover class for urban areas [48]. Alternatively, the user may generate urban/rural extents by classifying the population density layer (population_raster), or by uploading an urban/rural shapefile from another source. Choice of urban/rural boundary is highly dependent on the nature of the survey, as definitions of urban and rural populations differ across countries and disciplines [49]. The strata boundary raster (strata_raster) can be derived from administrative area boundaries, for example Map Library [50] or DIVA-GIS [51], though the user might upload alternative strata boundaries defining, for example, ecological regions or a program catchment area.

To select PSU seed cells, gs_sample classifies each cell in the gridded population dataset (population_raster) by urban or rural location (if cfg_sample_rururb = TRUE and urban_raster is specified), and assigns a stratum ID (strata_raster). Serpentine sampling is used such that cells are geographically ordered from west-to-east, north-to-south, and sampled based on a random starting cell and a population increment that produces the desired number of PSUs within the stratum, thus facilitating a randomized population-weighted sample. The user may halt the algorithm at this point leaving just one cell per PSU by setting the PSU growth parameter to false (cfg_psu_growth = FALSE).

If the PSU growth parameter is set to true (cfg_psu_growth = TRUE), gs_sample grows PSUs using a dilation filter routine to enlarge the area around each PSU seed cell by adding neighboring cells one cell at a time until the specified population per PSU parameter is met. From the seed cell, the dilation routine randomly chooses one of the nearest north, east, south, or west cells, and adds that population to the PSU. The routine loops over each PSU adding more population cells each time until each PSU achieves the maximum PSU area in square kilometers (cfg_max_psu_size) or total population per PSU value (cfg_pop_per_psu). A valid sample frame has contiguous, non-overlapping potential PSUs. Thus, *GridSample* restricts PSUs to being contiguous and non-overlapping by drawing voronoi polygons around each seed cell, defining unique areas in which each PSU can grow; the PSU growth routine will not add cells beyond a strata or voronoi polygon boundary.

After all PSUs have been selected, the algorithm generates a polygon shapefile of the PSU boundaries and assigns the following attributes to each PSU: PSU identifier, stratum identifier, urban/rural class of the seed cell, PSU centroid coordinate, total/urban/rural population in PSU, total/urban/rural population in stratum, number of cells in PSU, and number of PSUs in stratum (Table 1). The algorithm prints to screen the value of the random number used to start the sampling process; this value can be recorded and manually entered in *GridSample* to reproduce an existing sample. The following attributes are needed to calculate sample weights (presented later): number of selected PSUs in stratum (psus_in_stratum), estimated population in stratum (str_pop), and estimated population in PSU (psu_pop).

**Table 1:** Summary of attributes in the output shapefile

Label	Type	Description
PSUid	Integer	PSU identifier
stratum	Integer	Stratum identifier
psu_pop	Decimal	Estimated population in PSU derived by summing the seed cell and any growth cells selected for PSU
psu_r_pop	Decimal	Estimated rural population in PSU derived by summing all rural cells selected for PSU
psu_u_pop	Decimal	Estimated urban population in PSU derived by summing all urban cells selected for PSU
psus_in_stratum	Integer	Number of PSUs in the stratum
str_pop	Decimal	Estimated population in stratum derived by summing all grid cells
str_r_pop	Decimal	Estimated rural population in stratum derived by summing all grid cells classified as rural
str_u_pop	Decimal	Estimated urban population in stratum derived by summing all grid cells classified as urban
str_cells	Integer	Number of total cells in the stratum
xCent	Decimal	Longitude of PSU seed cell centroid in decimal degrees
yCent	Decimal	Latitude of PSU seed cell centroid in decimal degrees
U_R	Character	Urban or rural label based on whether the seed cell was classified as urban or rural

### *GridSample*: clustered sampling

*GridSample* supports the first-stage of the typical two-stage cluster design used by DHS, MICS, and LSMS, as well as several other common survey designs. The user defines the desired total population in each PSU (cfg_pop_per_psu), ranging from 400 to 600 people in typical household surveys. Alternatively, *GridSample* can be used to select one-stage cluster samples by setting the total population per PSU (cfg_pop_per_psu) equal to the number of households to be sampled per PSU (cfg_hh_per_urban and cfg_hh_per_rural) multiplied by the average household size (available from previous surveys). Likewise, *GridSample* might be used to select a random sample of households by setting total population per PSU (cfg_pop_per_psu) equal to the average household size, and setting the number of households to be sampled per PSU (cfg_hh_per_urban and cfg_hh_per_rural) equal to 1. To implement a random sample of households, the user would additionally need to use a method to identify a random dwelling within each PSU [8].

### *GridSample*: stratification

Strata should be mutually exclusive geographic areas that cover the entire population. In typical household surveys, sub-national administrative areas such as provinces or districts serve as strata, and sometimes these areas are further stratified into rural and urban areas. Independent samples will be selected from each stratum allowing strata-level estimates to be compared after the survey. While some gridded population datasets provide estimates of population by age-group and sex [25, 52, 53] or household poverty level [54, 55], *GridSample* does not currently include a mechanism for non-geographic stratification, though the user could, in principal, sample from gridded population datasets of social-demographic groups.

To generate a geographically stratified sample in *GridSample*, the user defines strata boundaries with strata_raster, and specifies the sample size per stratum with cfg_hh_per_stratum. This means that if the national sample size is 10,000 households from 5 provinces, then cfg_hh_per_stratum == 2000. If the survey were additionally stratified by urban/rural such that there are 10,000 households sampled from 10 strata, then strata_raster should include the boundaries of both urban/rural areas and provinces, and cfg_hh_per_stratum == 1000.

### *GridSample*: urban/rural oversampling

If urban/rural populations are not stratified, they may instead be treated as sub-domains. Sub-domains represent important sub-populations for which representative statistics are generated from the survey data, and thus each sub-domain should meet the minimum stratum sample size requirement (cfg_hh_per_stratum). If either the urban or rural sub-domain does not include enough households, then the algorithm uses the ordered data frame to choose the next cell from the under-represented sub-domain (from any strata) and swaps out an already selected seed cell of the opposite sub-domain within that stratum. This process repeats until the sample size requirement is met in each sub-domain (cfg_hh_per_stratum). To implement sub-domain representation in *gs_sample*, set cfg_sample_rururb == 1 and define urban/rural boundaries (urban_raster).

In practice, rural areas may be more difficult and expensive to visit, and thus a greater number of households might be sampled from rural PSUs than urban PSUs. This is why the user may specify different numbers of households to be sampled from urban PSUs (cfg_hh_per_urban) and rural PSUs (cfg_hh_per_rural). If the same number of households will be sampled from all PSUs, then the user only needs to specify households to be sampled from urban PSUs (cfg_hh_per_urban).

### *GridSample*: spatial oversampling and other features

Oversampling in space is analogous to oversampling urban/rural sub-domains. To select a sample that is both representative of the population and of space in *GridSample*, set cfg_sample_spatial == 1 and specify the spatial scale (in square kilometers) at which the sample should be representative (cfg_sample_spatial_scale). For example, cfg_sample_spatial_scale == 20 means that a coarse grid system with cells 20 km × 20 km will be overlaid on the study area. If a coarse grid cell does not contain a PSU seed cell, then the first cell within the serpentine ordered data frame located inside the course cell will be selected, and another seed cell from the same stratum and sub-domain will be randomly dropped. To overcome the issue of slightly smaller grid cells toward the poles, *GridSample* calculates the area of the centroid (geographic center) grid cell in the study area, and uses that average grid cell size to generate the coarse grid with the correct dimensions.

The spatial scale of the survey is ideally linked to the scale of planned small area estimates. For example, if the sample is stratified by province (level 1 administrate units), and small area estimates will later be generated for districts (level 2 administrative units), then the median size of districts could be used. Determining an appropriate spatial scale may take trial and error. If the country has large areas of sparse population, the user might need to (a) increase the size of the spatial scale (cfg_sample_spatial_scale), or (b) force the algorithm to generate more PSUs in each stratum by increasing the sample size per stratum (cfg_hh_per_stratum) and/or reduce the number of households sampled in each PSU (cfg_hh_per_urban and cfg_hh_per_rural).

*GridSample* offers several additional parameters. (1) The user can input a 100 m × 100 m gridded population dataset, and then aggregate cells for the sample frame (e.g. 300 m × 300 m sample frame cells would be generated by setting cfg_desired_cell_size = 3). Aggregating gridded population estimates usually increases the accuracy of each grid cell. Note that guidance regarding the ideal cell size of gridded population sample frames is not yet available. Other parameters include: (2) minimum population per cell (cfg_min_pop_per_cell) which will exclude grid cells from the sample frame with less than the specified minimum population, (3) maximum area of the PSU in squared kilometers (cfg_max_psu_size) to ensure that PSUs can be feasibly enumerated during fieldwork, (4) random number value (cfg_random_number) to reproduce a previous gridded population sample, and (5) halt the PSU growth process (cfg_psu_growth = FALSE) discussed in detail below.

## Results

We replicated the first-stage sample of the 2010 Rwanda DHS in *GridSample*. The 2010 Rwanda DHS sampled 12,540 households from 492 PSUs comprising rural villages and urban neighborhoods [56]. The sample was stratified by Rwanda’s 30 districts, urban areas were oversampled by adding 12 PSUs in Kigali’s three districts, and 26 households were sampled from each urban and rural PSU. The average village in Rwanda had 610 occupants according to the sample frame of 14,837 villages/neighborhoods. To replicate the 2010 Rwanda DHS in *GridSample*, we loaded the *GridSample* package, the *raster* package to prepare the data for *GridSample*, and set a working directory:
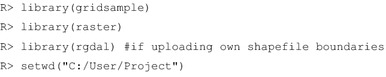


Next, we called the Rwanda 2010 UN-adjusted gridded population estimates preloaded in *GridSample* and also available at the WorldPop website [33]. This dataset was generated from 2002 Rwanda Census block data and 15 spatial covariates using a random forest model with dasymetric redistribution as described in the metadata [57] and cited methods paper [30].
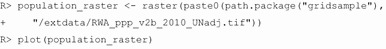


Then we loaded an unprojected shapefile of Rwanda’s 30 district boundaries to use as strata. This file is preloaded in *GridSample*, and can be downloaded from MapLibrary [50]. We rasterized strata boundaries using the WorldPop population raster dimensions and assigned strata ID (ADM2_ID) as the grid cell value (numeric district identifier values ranged from 1 to 30):
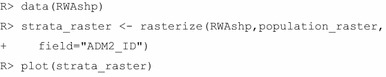


We considered using GUF, Modis or GRUMP to distinguish urban and rural areas, though we decided that these global models were not well suited for the largely rural context of Rwanda [38]. Instead, we calculated a sensible value to distinguish rural and urban cells directly from the WorldPop population raster. According to the 2012 Census, the National Institute of Statistics in Rwanda classifies 16% of the population as urban [58]. Thus, we identified the cell density value associated with 16% of the population living in the most populous cells, and used that value (11 people per 100 m × 100 m cell) to create a binary raster of urban areas (value 1) and rural areas (value 0).
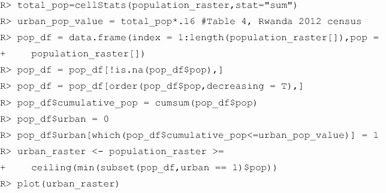


Note that the value used to differentiate urban and rural cells was found with the following code.
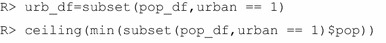


**Fig. 3:**
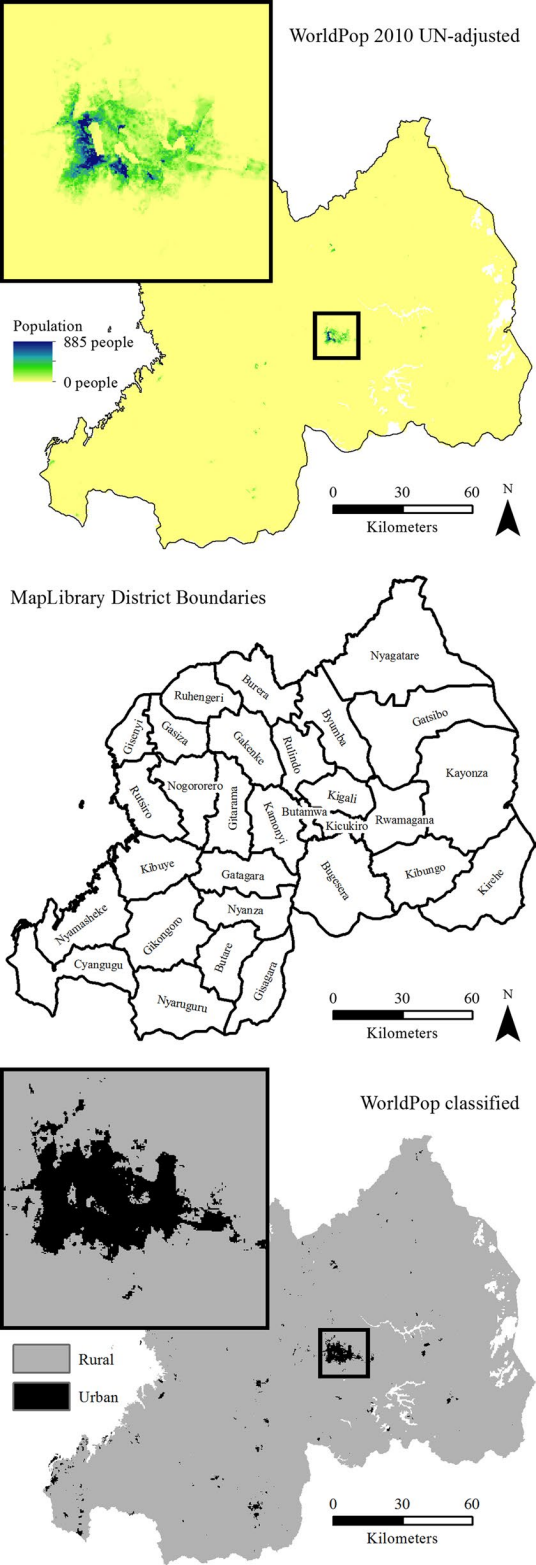
Input datasets to the Rwanda gridded population sample in *GridSample*

The gridded population, rasterized strata, and rasterized urban/rural layers are visualized in Fig. 3. We used these input data, plus parameters for total household sample size per stratum (cfg_hh_per_stratum = 416), grow PSUs (cfg_psu_growth = TRUE) to a minimum population total per PSU (cfg_pop_per_psu = 610), and household sample size per urban and rural PSU (cfg_hh_per_urban = 26 and cfg_hh_per_rural = 26), to generate a gridded population sample with the same design as the 2010 DHS. We prevented sampling of cells with very small probability of population (cfg_min_pop_per_cell = 0.01), limited the PSU size to 10 km × 10 km (cfg_max_psu_size = 10), and specified the name (sample_name = ”rwanda_psu_sample”) and file location (output_path = ” C:/User/Project/data”) to save the output shapefile.
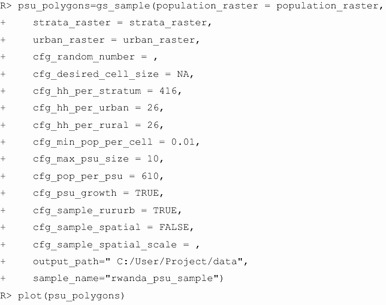

**Fig. 4:**
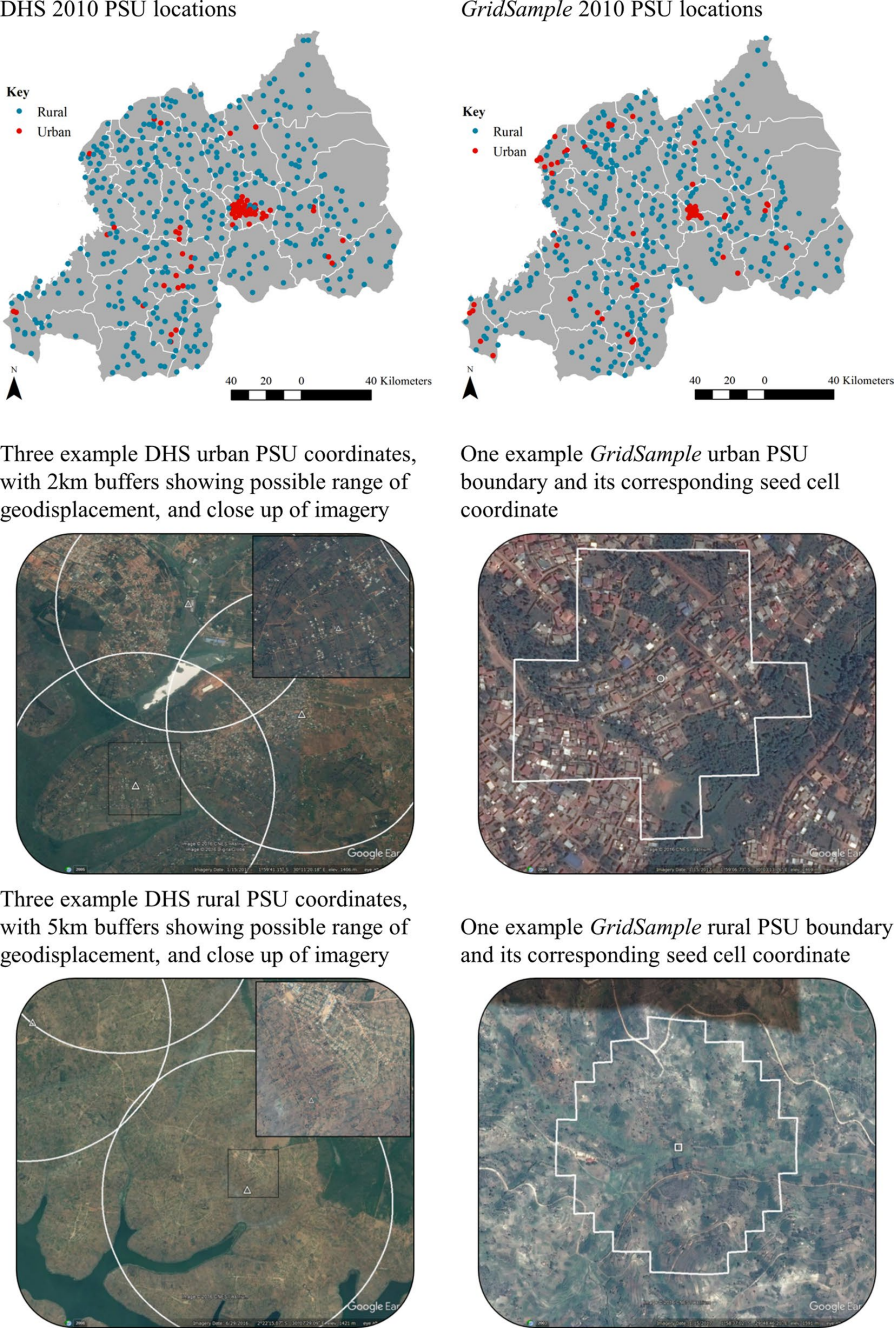
Visual comparison of primary sampling units (PSUs) generated by the 2010 Rwanda DHS [56] and *GridSample*

The Rwanda DHS selected 79 urban PSUs and 413 rural PSUs from their census sample frame. *GridSample* produced a similar sample of 75 urban PSUs and 405 rural PSUs (Table 2) which followed a similar geographic pattern as the Rwanda DHS (Fig. 4) using the WorldPop sample frame. In the *GridSample*-generated sample [59], the mean population per PSU was 620 people with one outlier that had 1479 people, and the median population was 612 people per PSU. One key difference between the samples was that the DHS added PSUs during the oversample, while *GridSample* re-distributed PSUs during the oversample, resulting in fewer PSUs. A second key difference was that DHS purposefully oversampled in the Kigali metropolitan area (Gasabo, Kicukiro and Nyarugenge districts) while *GridSample* oversampled from all urban areas, including smaller cities in Gisenyi, Cyangugu, and Gikongoro districts.

**Table 2:** Number of primary sampling units in a Demographic and Health Survey and equivalent *GridSample* survey

District name	Alternative name	DHS		*GridSample*	
		Urban	Rural	Urban	Rural
Bugesera	Bugesera		16	2	14
Burera	Burera		16	1	15
Butamwa	Nyarugenge	19	1	15	1
Butare	Huye	3	13	3	13
Byumba	Gicumbi	2	14	1	15
Cyangugu	Rusizi	2	14	5	11
Gakenke	Gakenke		16		16
Gasiza	Nyabihu		16	1	15
Gatagara	Ruhango	3	13		16
Gatsibo	Gatsibo		16		16
Gikongoro	Nyamagabe	1	15	3	13
Gisagara	Gisagara		16		16
Gisenyi	Rubavu	1	15	10	6
Gitarama	Muhanga	4	12	1	15
Kamonyi	Kamonyi		16		16
Kayonza	Kayonza		16		16
Kibungo	Ngoma	3	13	1	15
Kibuye	Karongi	2	14	2	14
Kicukiro	Kicukiro	20		13	3
Kigali	Gasabo	11	9	8	8
Kirehe	Kirehe		16		16
Nogororero	Ngororero		16		16
Nyagatare	Nyagatare		16		16
Nyamasheke	Nyamasheke		16		16
Nyanza	Nyanza	4	12	2	14
Nyaruguru	Nyaruguru		16		16
Ruhengeri	Musanze	2	14	3	13
Rulindo	Rulindo		16	1	15
Rutsiro	Rutsiro		16		16
Rwamagana	Rwamagana	2	14	3	13
Total		79	413	75	405

## Discussion

Gridded population sampling methods are in their infancy. Several approaches to first-stage sample selection and to fieldwork have been tried. These approaches are promising but have limitations and require further research. The *GridSample* R algorithm provides a tool to develop and evaluate emerging gridded population sampling methods.

### Modifiable Areal Unit Problem

Gridded population sampling is sensitive to the modifiable areal unit problem (MAUP). A MAUP emerges when an arbitrary spatial unit, such as a grid cell, is used to summarize continuous population characteristics leading to apparently different spatial patterns of that characteristic in the population simply by changing the size (scale) or zone (grouping) of the spatial units [60]. In gridded population sampling, the size and zone of grid cells are likely to influence sampling inclusion probabilities, especially when the first-stage sample is based on geographically large grid cells, and/or the population is heterogeneously distributed. 

**Fig. 5:**
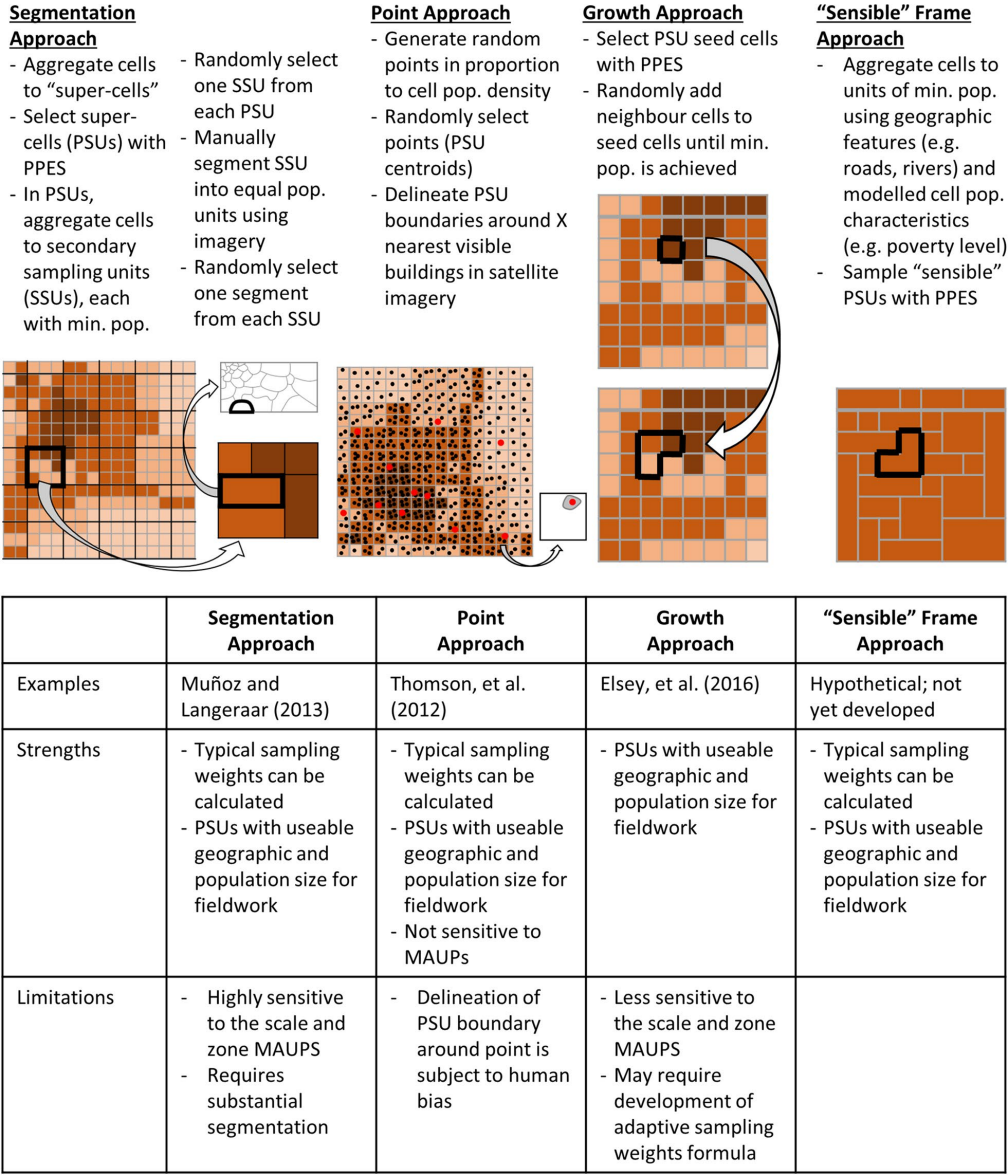
Probability sampling approaches with gridded population data

Four general approaches to first-stage sampling with gridded population data are outlined in Fig. 5. First, the segmentation approach involves sampling geographically large PSUs with probability proportionate to estimated population size, then segmenting by smaller grid cells [10] or manually delineate smaller areas using satellite imagery [6–10]. *GridSample* can be used to select large cells by aggregating the input gridded population dataset. In Myanmar, Muñoz and Langeraar (2013) aggregated LandScan 1 km × 1 km gridded population estimates to 3 km × 3 km “super” cells for selection of the first-stage sample. Then they grouped 1 km × 1 km grid cells within the selected PSUs to meet a minimum population threshold, and then randomly sampled one group of cells as a secondary sampling unit (SSU) in each PSU. Finally, they manually segmented SSUs into dozens of areas with roughly equal population based on satellite imagery, and sampled one segment [10]. As a result, the sample weights were computationally straightforward to calculate because they followed a typical multi-stage sampling approach. Additionally, the final sampling units had sensible boundaries related to features in the real world, making fieldwork feasible. However, sample inclusion probabilities of PSUs and SSUs were sensitive to the size and zone of grid cells, which could have smoothed-out or emphasized population density depending on the distribution of the underlying population.

A point approach was used by Thomson and colleagues (2012) using LandScan 1 km × 1 km gridded population data in the eastern D. R. Congo. For this survey, the team generated randomly located points within grid cells where the number of points was proportional to estimated population. Then they randomly sampled points within strata. Finally, they manually delineated sampling units around the nearest dwellings to each point using satellite imagery, ensuring that PSU boundaries were located within cell boundaries [6]. Sample weights were adapted to follow a typical multi-stage sampling approach, the final sampling unit boundaries were sensible, making fieldwork feasible, and the use of points prevented any effect of the MAUP. However, the manual delineation of one sampling unit around each point was subject to human bias.

The third approach to gridded population sampling is the growth approach, uniquely available in the *GridSample* tool. Elsey et al. [7] in Kathmandu, Nepal used an early version of *GridSample* to select seed cells from WorldPop’s 100 m × 100 m gridded population dataset, and grew PSUs to a minimum population size. Growing PSUs is likely less sensitive to the zone and scale MAUPs than segmenting large cells because, in the growth approach, the scale of the starting grid cells is closer in geographic and population size to the final sampling unit. However, the correct calculation of sampling inclusion probability weights for the growth approach is unclear. Should sample probability weights be calculated from the grid cell densities, or the densities of final sampling units? Arguments can be made for both approaches. Before discussing two potential sample weight calculations for the growth approach, we describe a hypothetical, but feasible, fourth approach to gridded population sampling.

Perhaps the most ideal gridded population sample frame would group grid cells into “sensible” potential PSUs of similar population size before first-stage sampling. Sensible PSU boundaries would be defined in terms of geographic features such as roads, rivers, ridges or valleys that could be easily recognized and navigated in the field. Sensible PSUs would also group similar types of populations, for example, by grid cell mean poverty level. Generation of a sensible gridded population sample frame has only recently become possible as new techniques are developed to estimate population characteristics, such as poverty-level or disease status, in a gridded population format [54, 61]. The use of quadtree methods to divide dense population grid squares into four smaller cells can be viewed as a rudimentary first step toward development of sensible potential PSUs [62]. If a gridded population sample frame of sensible potential PSUs existed, the survey practitioner would sample units with probability proportionate to estimated size, and calculate typical sampling inclusion probability weights.

The growth approach to gridded population sampling may be conceptualize of as one instance of a sensible frame in which only the boundaries of the sampled PSUs are known, and the boundaries of non-sampled potential PSUs exist but are not drawn. Sample weights calculated from the final PSU population densities are straightforward to calculate, and are provided below.

If, however, the growth approach inclusion probabilities need be calculated from grid cell (rather than final PSU) population densities, then a complex adaptive sample weight needs to be formulated [63]. An adaptive sample weight would account for the estimated population of a given cell, as well as the probability of being grown into a PSU via a neighboring cell. The probability of being grown into a PSU would depend on (a) the estimated populations of neighboring cells, (b) the parameter for PSU maximum geographic size, (c) the parameter for PSU minimum population size, and possibly (d) the location of strata boundaries, and (e) the location of voronoi polygon boundaries between seed cells in a multitude of sample instances. The need for such a complex formulation needs to be evaluated, but is beyond the scope of this paper.

### Sample weights

Below, we provide sample weight calculations for the growth approach to PSU selection, which is uniquely available in *GridSample*. These weights reflect inclusion probabilities in the final PSUs, and not of individual grid cells. Sample weights for the segmentation, point, and sensible PSU approaches have been described elsewhere and are summarized in Additional file 1. These sample weight formulations parallel typical survey methods, reflecting the probability that a household is (1) selected, (2) found, and (3) responded [13–16]. While the *GridSample* output shapefile includes values needed to calculate PSU selection probabilities, the survey implementer must track the number of households enumerated in the field in each PSU, and household response rates to correctly calculate sample weights. The following formulas use four indices: 1…*k* strata, 1…*i* PSUs, 1…*j* households, and 1…*q* individuals. The household selection (base) weight for the growth approach to PSU formation—the probability that PSU *i* is selected, and then household *j* is selected—is given by:

1 $$w_{ij.b} = \frac{1}{{P_{i} \times P_{j\left( i \right)} }} = \frac{{G_{k} /g_{ik} }}{{n_{k} }} \times \frac{{M_{ik} }}{{m_{ik} }}$$

where $n_{k}$ is the number of selected PSUs in stratum *k*, $G_{k}$ is the estimated total population in stratum *k*, $g_{ik}$ is the estimated population in PSU *i* in stratum *k*, $m_{ik}$ is the number of households sampled in PSU *i* and stratum *k* during fieldwork, and $M_{ik}$ is the number of total households enumerated in PSU *i* and stratum *k* during fieldwork.

If growth PSUs are manually divided and further sampled, weights are calculated in the same way, except that the probability of being in the final sample unit $w_{ij.b}$ includes $b_{ik}$, the proportion of households located in the manually-drawn segment, approximated by counting buildings in satellite imagery:

2 $$w_{ij.b} = \frac{{G_{k} /g_{ik} }}{{n_{k} }} \times \frac{{M_{ik} }}{{m_{ik} }} \times \frac{1}{{b_{ik} }}$$

The household response weight—the probability that PSU *i* is found and sampled, and household *j* is found and responded—is given by:

3 $$w_{ij.r} = \frac{1}{{P_{i.r} \times P_{j.r\left( i \right)} }} = \frac{{n_{k} }}{{n_{k} *}} \times \frac{{m_{ik} }}{{m_{ik} *}}$$

where $n_{k}$ number of selected PSUs in stratum *k*, $n_{k} *$ is the number of found and sampled PSUs in stratum *k*, $m_{ik}$ is the number of selected households in PSU *i* and stratum *k*, and $m_{ik} *$ is the number of found and responded households in PSU *i* and stratum *k*. The individual response weight—the probability that PSU *i* is found and sampled, then household *j* is found and responds, and finally that individual *q* is present and responds—is given by:

4 $$w_{ijq.r} = \frac{1}{{P_{i.r} \times P_{j.r\left( i \right)} \times P_{{q.r\left( {ji} \right)}} }} = \frac{{n_{k} }}{{n_{k} *}} \times \frac{{m_{ik} }}{{m_{ik} *}} \times \frac{{u_{ijk} }}{{u_{ijk} *}}$$

where $n_{k}$ is the number of selected PSUs in stratum *k*, $n_{k} *$ is the number of found and sampled PSUs in stratum *k*, $m_{ik}$ is the number of selected households in PSU *i* and stratum *k*, $m_{ik} *$ is the number of found and responded households in PSU *i* and stratum *k*, and $u_{ijk}$ is the number of eligible individuals in household *j* in PSU *i* and stratum *k*, and $u_{ijk} *$ is the number of responded individuals in household *j* in PSU *i* and stratum *k*. The household sample weight $w_{ij}$ is comprised of the household selection weight and household response weight:

5 $$w_{ij} = w_{ij.b} \times w_{ij.r}$$

Assuming that all eligible individuals (e.g., all women age 15–49) will be interviewed in the selected households, the individual sample weight $w_{ijq}$ is comprised of the household selection weight and individual response weight:

6 $$w_{ijq} = w_{ij.b} \times w_{ijq.r}$$

### Fieldwork

**Fig. 6:**
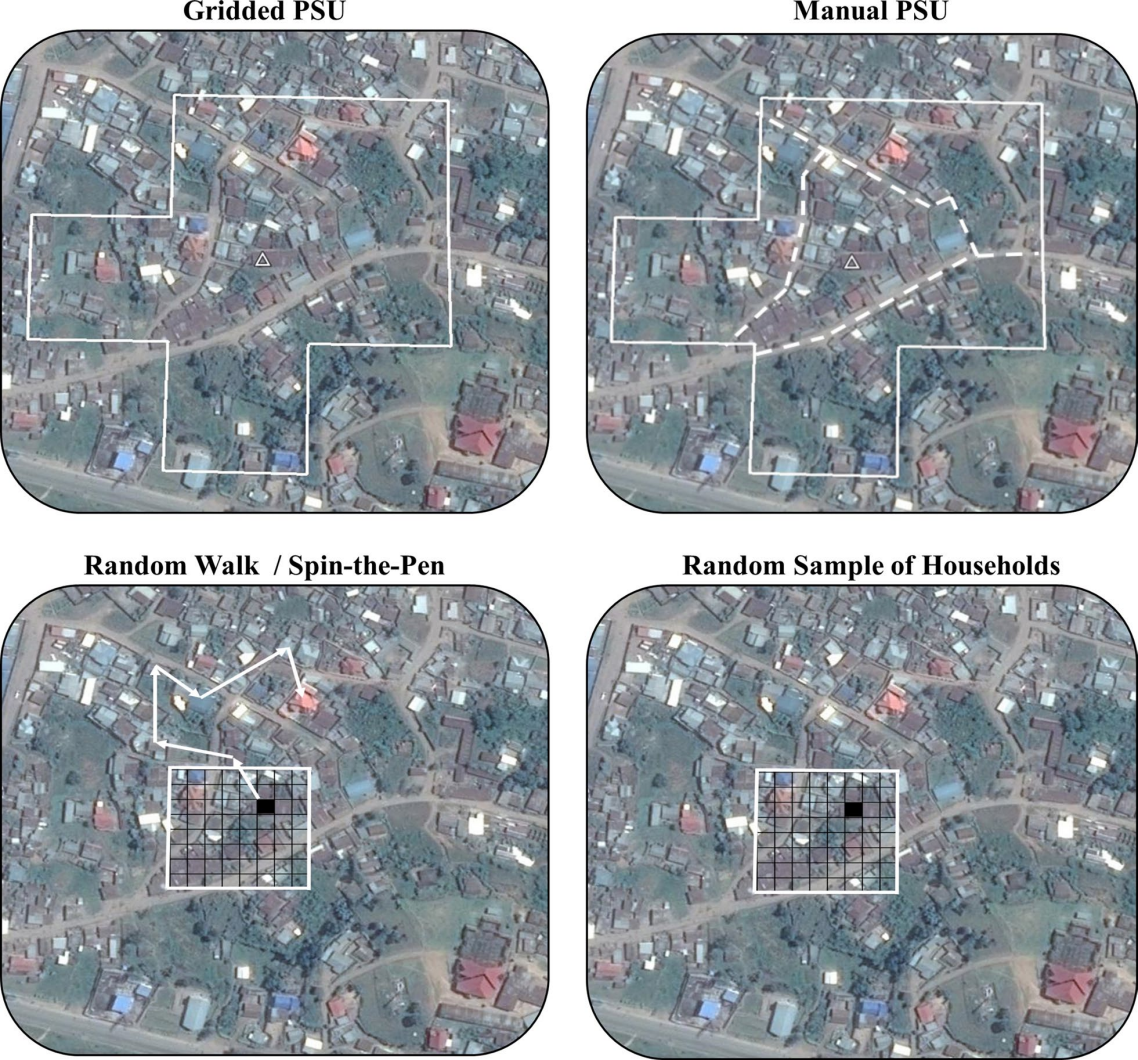
Schematic of four field implementation options for gridded population sampling

Four approaches are available for survey fieldwork with *GridSample* output. These four approaches are visualized in Fig. 6, and described below.

#### Gridded PSUs

This option uses gridded PSU boundaries which have squared corners and no relation to geographic or administrative features in the real world. This approach was used in a two-stage cluster survey of households in Kathmandu, Nepal [7]. The team used OpenStreetMap™, a crowd sourced online map of roads, building locations, and other features, via an Android application on mobile phones to digitally map households within PSUs. OpenStreetMap™ enumeration was chosen over typical pen-and-paper mapping, in part, because half of their PSUs were already mapped in OpenStreetMap™. Households (defined as a group of people who share a cook pot) were fully enumerated by knocking on doors and talking to neighbors ensuring that lower-income households who shared an apartment were not under-sampled. The team encountered, sometimes substantial, differences in the number of households per PSU than were expected from the WorldPop sample frame, so they planned to interview every 10^th^ household regardless of PSU size to achieve a probability sample. The team cited geographic accuracy in field maps, feasibility of mapping in dense, complex urban environments, leveraging of existing data, and the ability to contribute to a crowd-sourced resource as reasons to use this approach [7].

We support the use of OpenStreetMap™ enumeration, especially for urban settings where OpenStreetMap™ data are likely to exist. However, we strongly recommend that implementers employ a method to anonymize buildings added to the crowd-sourced map such that interviewed PSUs cannot be identified. In areas where buildings have already been mapped in OpenStreetMap™, minor edits will not reveal PSU locations. However, in areas of the map without building and road locations, implementers should consider mapping beyond the edges of the PSU boundaries so that gridded PSU shapes do not suggest a gridded household survey. Furthermore, if OpenStreetMap™ data are sparse in the survey region, implementers should consider enumerating a number of fake PSUs to preserve the anonymity of interviewed communities. Specific guidelines for OpenStreetMap™ enumeration are not yet available.

#### Manually-drawn PSUs

 A second approach to implement gridded population samples is to manually draw PSUs around random points within seed cells, or to manually segment gridded sampling units using detailed satellite imagery. Manually-drawn PSUs were used in a one-stage cluster survey in eastern D. R. Congo [6] and a two-stage cluster survey in Myanmar [10]. A key benefit of this approach is that PSUs follow sensible boundaries such as rivers and roads, which are easily identified in both satellite images and in the field. Because manually-drawn PSUs are easily identifiable, field teams are flexible to use hand-sketched pen and paper maps, printed maps of satellite imagery or OpenStreetMap™ features, or digital maps for field navigation and household enumeration.

#### Non-probability samples

Random-walk and “spin-the-pen” sampling methods result in non-probability samples of the population and are thus not recommended by surveyors [64–66]. Nonetheless, these and similar methods are often used in rapid or high-security field assessments because they are cheaper and faster to implement than typical two-stage cluster samples. Random-walk and spin-the-pen gridded sampling methods were used in rapid assessments in Iraq [8] and Myanmar [11]. In both studies, gridded population datasets were considered to be more accurate sample frames than other available population data. Because random-walk and spin-the-pen methods do not lead to probability samples, we do not provide sample probability weights.

#### Simple random sample of households

Researchers sometimes perform simple random samples of households in small study areas—for example, a refugee camp or a single city—by digitizing dwelling point locations in a satellite image and sampling points at random [67–71]. While a simple random sample of households has not been conducted using gridded population sampling, it would be straightforward to implement. Grid cells would be sampled with probability proportionate to estimated size, and the growth algorithm could optionally be switched off to generate single cell PSUs. Then a single dwelling would be randomly chosen within selected cells, either from mapping all dwellings or using a method like the one described by Galway and colleagues in Iraq [8]. In the Iraq study, the team overlaid a 10 m × 10 m mini-grid on Google Earth™ satellite imagery within the seed cell, and then randomly selected one mini-grid unit. If the 10 m mini-grid unit covered a building, the building was selected for sampling, otherwise the process was repeated until the first building was randomly identified in the imagery. If the randomly selected building had multiple households or was non-residential, one nearby household could be randomly selected as describe by Siri and colleagues in Kenya [66]. A simple random sample of households would not require sample weights.

### Limitations

Gridded population data are increasingly used as an alternative survey sample frame in countries where census data are outdated or inaccurate. Gridded population sample frames may also be used in lieu of census data for surveys that need to be representative of both population and of space, and where PSUs of a specific population size are needed. Next we discuss six areas where research is underway, or needed, to address limitations of gridded population sampling.

#### Accuracy of gridded population sample frame

The first major concern in gridded population sampling is the accuracy of the underlying gridded population data. Gridded population sampling has been tried by a number of survey implementers in circumstances of outdated or inaccurate census data, however the accuracy of gridded population datasets are varied, and often unquantified. Accuracy of publically available top-down gridded population data is dependent on several model components: (1) accuracy of the input census data, (2) the geographic scale of the input census data (e.g. census tract-level versus district-level), (3) the age, accuracy, and type of model covariate data, and (4) the model algorithm itself. The geographic scale of the output grid also matters for measurement of accuracy; grid cell estimates in a 1 km × 1 km gridded population dataset will almost always be more accurate than grid cells in a 100 m × 100 m gridded population dataset. Model errors are difficult to estimate, and to even conceptualize, for gridded population datasets that rely on simple disaggregation approaches, as they are essentially gridded representations of the input census data [24]. While prediction errors can be calculated for gridded population datasets derived from complex modelling techniques, WorldPop is the only dataset to include errors [see, for example, 56]. However, it is unclear how survey implementers can use prediction errors to quantify or improve the accuracy of household survey sample frames.

Numerous studies have evaluated the accuracy of gridded population estimates against ground-collected settlement locations [72], against census data available at a finer-scale than the census data used in the model [29, 73–76], and by comparing old and new gridded population datasets where the new dataset uses updated or finer-scale population data [38]. Still this evidence is not sufficient to assess the accuracy of a specific top-down gridded population dataset. Given the number of components that contribute to gridded population model error, future research should utilize simulation studies to test the effects of various model components on gridded population estimates. These studies should also reframe how the estimate errors are addressed (e.g. rather than ask “how much error is there around the estimate for each cell of size X?”, researchers should ask “how many cells need to be aggregated to achieve an error of Y?”).

#### Modifiable areal unit problem

Second, segmentation and growth approaches to sample unit selection might be subject to bias from the MAUP. Simulation studies should be used to quantify the effects of grid cell sizes and groupings on PSU selection probabilities. Additionally, development of geographically and socially sensible sample frames with gridded population data should be pursued. The ability to create a sensible gridded population sample frame is highly dependent on availability of fine scale, accurate environmental data and gridded estimates of population social-health characteristics.

#### Adaptive PSU sample weights

Third, where growth approaches are used for selection of PSUs, further research is needed to evaluate whether adaptive sample weights should be used, and if so, how to formulate them. These questions can be evaluated with statistical theory and simulation studies.

#### Availability of satellite imagery

Fourth, all of the approaches to gridded population sampling described here are dependent on access to fine-resolution satellite imagery with good visibility of dwellings without extensive tree-cover or cloud-cover. Existing gridded population samples have been implemented in cities, camps, deserts, savannah, and deforested farmlands; methods for implementing gridded population samples have not been described for forested areas.

#### Concealing PSU locations in publications and crowd-sourced maps

Fifth, gridded population samples that use crowd-sourced maps in fieldwork must guarantee anonymity of survey respondents and their communities. Crowd-sourced maps can be incredibly valuable for field navigation and household enumeration, though the technology and protocols to support survey activities are limited. Standard protocols have not yet been established to conceal survey PSU locations when mapping buildings and roads in a crowd-sourced platform such as OpenStreetMap™. Furthermore, we are not aware of any applications that allow survey enumerators to both update OpenStreetMap™ and separately store a confidential household listing linked to building locations, which interviewers would need to identify sampled households. As in any survey, PSU boundaries and centroid point locations should not be shared publically to protect the anonymity of respondents and their communities. PSU point locations can be published if they are randomly geo-displaced following methods like those used by MeasureDHS [77]. The MeasureDHS project publishes PSU centroid coordinates that are displaced up to 2 km in urban areas, and up to 5 km in rural areas, with one in every 100th rural point displaced up to 10 km.

#### Fieldwork feasibility

The sixth concern of gridded population sampling is feasibility of fieldwork. While there are multiple reasons to use gridded population sampling, protocols to use these methods in the field need further development. What is the enumeration protocol in a PSU that falls on two sides of a river where there is not a nearby bridge to cross? Should buildings be enumerated if they are intersected by the PSU boundary? Given that gridded PSU boundaries do not follow sensible geographic or administrative boundaries, recent satellite imagery is almost certainly needed during enumeration. What is the minimum image resolution required for sampling in rural versus urban areas? How recent should the satellite imagery be? What are the tradeoffs of using digital enumeration methods over paper-based methods? While the use of smart phones or tablets to digitally enumerate PSUs increases the cost and skill requirements among enumerators, it may also reduce the time and increase the accuracy of enumeration compared to pen-and-paper methods. Multiple issues related to cost, time, accuracy, technology, and staff skill requirements to implement gridded population surveys need to be evaluated.

## Conclusions

 The *GridSample* R package facilitates further research into the promising field of gridded population sampling. Gridded population sampling is an attractive alternative to typical sampling methods when census data are outdated or inaccurate. *GridSample* supports standard complex survey designs including clustered sampling, stratification, and oversampling in urban or rural areas. *GridSample* additionally allows users to oversample in space, and to specify a desired population size of sampling units. We show that *GridSample* can be used to replicate a DHS in Rwanda, providing evidence of a similar number of primary sampling units with similar population sizes in urban and rural areas. We also summarize four ways in which gridded population samples have been implemented in the field, and provide sample weight calculations for *GridSample* output. Finally, we discuss several areas of current and future research into gridded population sampling which can benefit from this tool.

## Additional file

**Additional file 1.** Sample weight formulas for segmentation, point, and sensible PSU formation.

## Abbreviations

CRAN: Comprehensive R Archive Network
DHS: Demographic and Health Survey
EA: Enumeration area
GHSL: Global Human Settlement Layer
GPW: Gridded Population of the World
GRUMP: Global Rural–Urban Mapping Project
GUF: Global Urban Footprint
LSMS: Living Standards Measurement Survey
MAUP: modifiable areal unit problem
MICS: Multiple Indicator Cluster Survey
PPES: probability proportionate to estimated size
PSU: primary sampling unit
SDG: sustainable development goals
SSU: secondary sampling unit
UNEP: United Nations Environment Programme
