# Supplementary material for: GridSample: an R package to generate household survey primary sampling units (PSUs) from gridded population data
Source: Int J Health Geogr. 2017 Jul 19;16:25. doi: 10.1186/s12942-017-0098-4 (PMC5518145; doi:10.1186/s12942-017-0098-4)
Supplement: Supplementary file 1 — Additional file 1. Sample weight formulas for segmentation, point, and sensible PSU formation. [file 12942_2017_98_MOESM1_ESM.docx]

**Additional file 1. Sample weight formulas for segmentation, point, and sensible PSU formation**

***GridSample*: An R package to generate household survey primary sampling units (PSUs) from gridded population data**

#### “Sensible” approach to PSU formation

If a gridded population sample frame of sensible potential primary sampling units (PSUs) existed, the survey practitioner would sample potential PSUs with probability proportionate to estimated size, resulting in primary sampling units (PSUs), and calculate typical sample inclusion probability weights. The following formulas use four indices: 1…*k* strata, 1…*i* PSUs, 1…*c* cells, 1…*j* households, and 1…*q* individuals. The household selection (base) weight – the probability that PSU *i* is selected, and then household *j* is selected – is given by:

|  | $w_{ij.b}=\frac{1}{P_{i}\times P_{j(i)}}=\frac{N_{k}}{n_{k}}\times\frac{M_{ik}}{m_{ik}}$ | (1) |
| --- | --- | --- |

where $N_{k}$ is the total number of potential PSUs in stratum *k*, $n_{k}$ is the number of selected PSUs in stratum *k*, $m_{ik}$ is the number of households sampled in PSU *i* and stratum *k* during fieldwork, and $M_{ik}$ is the number of total households enumerated in PSU *i* and stratum *k* during fieldwork.

#### Segmentation approach to PSU formation

The segmentation approach to PSU development involves sampling geographically large PSUs with probability proportionate to estimated population size, then segmenting PSUs by smaller grid cells ^[[1]](#footnote-1)^ and/or manually delineating secondary and even tertiary sampling units using satellite imagery^1,^^[[2]](#footnote-2)^.

In Myanmar, for example, Muñoz and Langeraar (2013) aggregated LandScan 1 kilometer X 1 kilometer gridded population estimates to 3 kilometer X 3 kilometer “super” cells for selection of the first-stage sample^1^. Then they grouped 1 kilometer X 1 kilometer grid cells within the selected PSUs to meet a minimum population threshold, and then randomly sampled one group of cells as a secondary sampling unit (SSU) in each PSU. Finally, they manually segmented SSUs into dozens of areas with roughly equal population based on satellite imagery, and sampled one segment (tertiary sampling unit – TSU). Although the team did not report sample weight calculations, sample weights could be easily calculated as follows.

|  | $w_{ij.b}=\frac{1}{P_{i}\times P_{j(i)}}=\frac{N_{k}}{n_{k}}\times\frac{1}{b_{ik}}\times\frac{1}{s_{ik}}\times\frac{M_{ik}}{m_{ik}}$ | (2) |
| --- | --- | --- |

where $N_{k}$ is the total number of potential PSUs in stratum *k*, $n_{k}$ is the number of selected PSUs in stratum *k*, $b_{ik}$ is the proportion of households located in the sampled cell (SSU) within “super cell” PSU *i*, $s_{ik}$ is the proportion of households located in the sampled segment (TSU) within SSU *I,* $m_{ik}$ is the number of households sampled in PSU *i* and stratum *k* during fieldwork, and $M_{ik}$ is the number of total households enumerated in PSU *i* and stratum *k* during fieldwork.

**Point approach to PSU formation**

A point approach was used by Thomson and colleagues (2012) using LandScan 1 kilometer X 1 kilometer gridded population data in the eastern D. R. Congo^2^. For this survey, the team generated randomly located points within grid cells where the number of points was proportional to estimated population. Then they randomly sampled points within strata. Finally, they manually delineated sampling units around the nearest dwellings to each point using satellite imagery, ensuring that PSU boundaries were located within cell boundaries. The team reported the household (base) sample weight calculation as follows.

|  | $w_{cj.b}=\frac{1}{P_{c}\times P_{j(c)}}=\frac{N_{k}}{n_{ck}*n_{k}}\times\frac{P_{ck}/h_{ck}}{m_{ck}}$ | (3) |
| --- | --- | --- |

where $N_{k}$ is the total number of potential PSUs (points) in stratum *k*, $n_{c}$ is the number of selected PSUs (points) in cell *c* and stratum *k*, $n_{k}$ is the number of selected PSUs (points) in stratum *k*, $m_{ck}$ is the number of households sampled in cell *c* and stratum *k* during fieldwork, $P_{ck}$ is the total estimated population in cell *c* in stratum *k* (from the gridded dataset), and $h_{ck}$ is the average household size in cell *c* and stratum *k* determined during fieldwork.

1. Muñoz J, Langeraar W. A census-independent sampling strategy for a household survey in Myanmar. 2013. http://winegis.com/images/census-independent-GIS-based-sampling-strategy-for-household-surveys-plan-of-action removed.pdf. Accessed 10 Mar 2017. [↑](#footnote-ref-1)
2. Thomson DR, Hadley MB, Greenough PG, Castro MC. Modelling strategic interventions in a population with a total fertility rate of 8.3: a cross-sectional study of Idjwi Island, DRC. BMC Public Health. 2012; doi:10.1186/1471-2458-12-959. [↑](#footnote-ref-2)
